# Risk of atrial fibrillation in patients with pneumoconiosis: A nationwide study in Taiwan

**DOI:** 10.1002/clc.23290

**Published:** 2019-11-29

**Authors:** Wei‐Syun Hu, Cheng‐Li Lin

**Affiliations:** ^1^ School of Medicine, College of Medicine China Medical University Taichung Taiwan; ^2^ Division of Cardiovascular Medicine, Department of Medicine China Medical University Hospital Taichung Taiwan; ^3^ Management Office for Health Data China Medical University Hospital Taichung Taiwan

**Keywords:** atrial fibrillation, cohort, pneumoconiosis

## Abstract

**Background:**

To investigate the incidence of new‐onset atrial fibrillation (AF) among subjects with pneumoconiosis using the Taiwan National Health Insurance Research Database.

**Hypothesis:**

Pneumoconiosis patients are at an increased risk of AF.

**Methods:**

A total of 12 209 pneumoconiosis patients were in the exposure cohort. Patients without pneumoconiosis were included as the comparison cohort. Both cohorts were matched by gender, age, comorbidity, and index year in a 1:1 manner. Multivariable cox proportional hazard model was used to calculate the adjusted hazard ratios (HRs) after adjustment for age, sex, and all comorbidities.

**Results:**

The risk of AF in pneumoconiosis patients was 1.30‐fold higher than that of controls (95% CI = 1.17‐1.44) was the key finding.

**Conclusions:**

Pneumoconiosis is associated with increased risk of incident AF.

## 1 INTRODUCTION

The importance of atrial fibrillation (AF) is increasing recognized since the impact of AF on thromboembolism, adverse cardiovascular outcomes and mortality has been evaluated and well documented.^1^, ^2^, ^3^ Indeed, CHA2DS2VASC score, which contained several critical comorbidities, is reported be a good predictor of poor outcomes in AF patients.^4^, ^5^, ^6^, ^7^

Pneumoconiosis is a chronic condition with multiple triggering factors and includes multiple clinical presentations.^8^, ^9^, ^10^, ^11^, ^12^, ^13^ Moreover, the course can be influenced by permanent exposure to toxins, including tobacco.^8^, ^9^, ^10^, ^11^, ^12^, ^13^ Indeed, pneumoconiosis, one kind of environmental lung disease, has been postulated across a number of clinical studies to commonly coexist with cardiovascular diseases.^8^, ^9^, ^10^, ^11^, ^12^, ^13^ However, there is no data presented regarding pneumoconiosis and AF. Using a large administrative database in Taiwan, we argued whether this association exists and to explore whether pneumoconiosis is a risk factor for (or “an independent predictor of”) AF.

## 2 METHODS

### 2.1 Data source

All data were acquired from the National Health Insurance Research Database (NHIRD), which was established by National Health Research Institutes and insured over 99% residents in Taiwan.^14^ The database contained abundant health and medical treatment information of the insurant. In the present study, we used inpatient file to explore the association between pneumoconiosis (ICD‐9‐CM code 500, 501, 502, 503, and 505) and AF (ICD‐9‐CM code 427.31). We further confirmed the diagnosis of pneumoconiosis using Registry of Catastrophic Illness Patient Database (RCIPD), which was a subset of the NHIRD. RCIPD contained the insurant who met the qualifications of catastrophic illnesses card (30 categories of diseases requiring long‐term care, such as liver cirrhosis with complication, chronic renal failure, malignant neoplasm, and chronic mental illness). A rigorous laboratory, pathological examination and clinical diagnosis is evaluated before the patient is issued with a Catastrophic Illness Certificate (CIC). RCIPD contained demographic data (including sex and date of birth ) and relevant information regarding CIC status (including category of catastrophic illness, date of diagnosis, and so on). Besides, all history diagnoses in the database were coded according to the International Classification of Disease, Ninth Revision, Clinical Modification (ICD‐9‐CM). The Research Ethics Committee of China Medical University and Hospital in Taiwan approved the study (CMUH‐104‐REC2‐115‐R3).

### 2.2 Study population

The exposure cohort was enrolled from RCIPD with a diagnosis of pneumoconiosis during 2000 to 2011, and the diagnosis date was defined as the index date. We excluded patients who were younger than 20 years old or who had preexisting AF prior to the index date.

A total of 12 209 pneumoconiosis patients were in the exposure cohort.

Patients without pneumoconiosis were included as the comparison cohort. The comparison cohort was collected using the same criteria as exposure cohort and individually matched by gender, age, comorbidity, and index year in a 1:1 manner. The endpoint of follow‐up was the date of AF diagnosis, death, withdrawal from NHI, or December 31, 2013, whichever came first. Comorbidities included hypertension, diabetes mellitus, hyperlipidemia, coronary artery disease (CAD), peripheral arterial occlusive disease (PAOD), chronic obstructive pulmonary disease (COPD), heart failure, asthma, interstitial lung disease, bronchiectasis, tuberculosis, liver cirrhosis, cancer, chronic kidney disease, hyperthyroidism, gout, sleep disorders, and stroke.

### 2.3 Statistical analysis

The statistical differences between two cohorts were determined through the chi‐square test for categorical variables and *t* test for continuous variables, respectively. Cox's proportional hazards model were used to evaluate the hazard ratio (HR) for exploring the association between pneumoconiosis and AF. Multivariable cox proportional hazard model was used to calculate the adjusted HRs after adjustment for age, sex, and all comorbidities. Analysis of stratification by age, gender, and comorbidity was performed to explore the association between pneumoconiosis and AF among the specific population. The cumulative incidence of AF was estimated using the Kaplan‐Meier method, and a log‐rank test was used to compare the incidence curves of the exposure cohort and comparison cohort. In this study, SAS 9.4 (SAS Institute, Inc., Cary, NC) was used for statistical analysis and the results were considered statistically significant if the two‐tailed *P*‐values were < .05.

## 3 RESULTS

**Figure 1:**
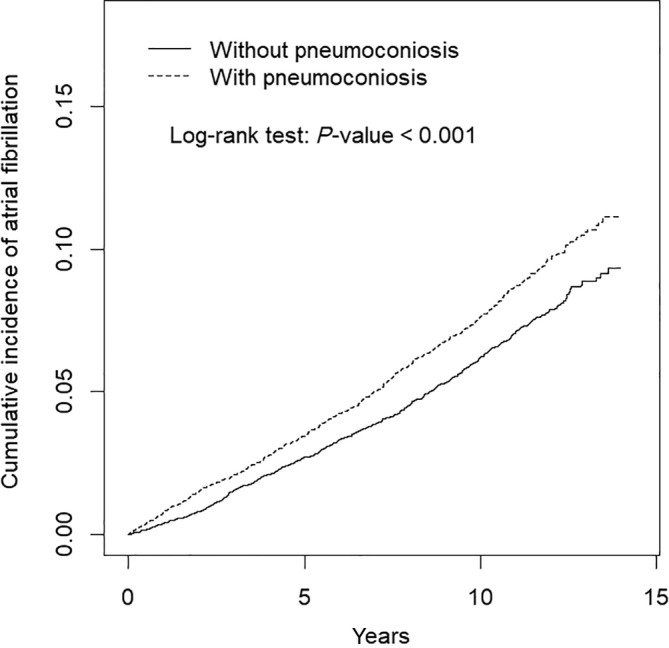
Cumulative incidence of atrial fibrillation for patients with and without pneumoconiosis

The mean (SD) of follow‐up in years was 8.12 (4.21) for exposure cohort and 8.76 (3.99) for comparison cohort. Distribution of baseline characteristics was described as number and percentage and is shown in Table 1. The mean (SD) age was 68.4 (7.06) and 68.4 (7.4) in the exposure cohort and comparison cohort, respectively. There were 88% male and 11% female in the study cohort. Comorbidities had no difference between exposure cohort and comparison cohort except for chronic kidney disease (*P* = .03). The exposure cohort had a significant higher cumulative incidence rate of AF than comparison cohort with a log‐rank test *P* < .001 (Figure 1).

**Table 1:** Characteristics of patients with pneumoconiosis and matched subjects without pneumoconiosis

	Pneumoconiosis				
	Yes		No		
	(N = 12 209)		(N = 12 209)		
	n	%	n	%	*P*‐value
Age, year					.64
≤64	3813	31.2	3786	31.0	
65‐74	6370	52.2	6342	52.0	
≥75	2026	16.6	2081	17.0	
Mean (SD)	68.4	7.06	68.4	7.40	.98
Gender					0.50
Female	1393	11.4	1427	11.7	
Male	10 816	88.6	10 782	88.3	
Comorbidity					
Hypertension	5367	44.0	5370	44.0	0.97
Diabetes mellitus	2609	21.4	2598	21.3	0.86
Hyperlipidemia	1290	10.6	1247	10.2	0.37
Coronary artery disease (CAD)	2919	23.9	2877	23.6	0.53
Peripheral arterial occlusive disease (PAOD)	328	2.69	310	2.54	0.47
Chronic obstructive pulmonary disease (COPD)	4697	38.5	4739	38.8	0.58
Heart failure	1507	12.3	1418	11.6	0.08
Asthma	1770	14.5	1699	13.9	0.19
Interstitial lung disease	26	0.21	21	0.17	0.47
Bronchiectasis	4746	38.9	4790	39.2	0.56
Tuberculosis	1945	15.9	1909	15.6	0.53
Liver cirrhosis	1513	12.4	1465	12.0	0.35
Cancer	1924	15.8	1867	15.3	0.31
Chronic kidney disease	535	4.38	468	3.83	0.03
Hyperthyroidism	46	0.38	41	0.34	0.59
Gout	1384	11.3	1359	11.1	0.61
Sleep disorders	488	4.00	447	3.66	0.17
Stroke	2574	21.1	2598	21.3	0.71

Pneumoconiosis was significantly associated with AF with a 1.30‐fold increased risk. Among male subjects, compared to comparison cohort, those with pneumoconiosis had multivariate HR (95% CI) for AF of 1.33 (1.19‐1.48). Among subjects younger than 64 years, compared to comparison cohort, those with pneumoconiosis had multivariate HR (95% CI) for AF of 1.58 (1.23‐2.03). Among subjects aged 65‐74 years, compared to comparison cohort, those with pneumoconiosis had multivariate HR (95% CI) for AF of 1.26 (1.10‐1.44). Among subjects without any comorbidities, compared to comparison cohort, those with pneumoconiosis had multivariate HR (95% CI) for AF of 1.70 (1.35‐2.14). Among subjects with any comorbidities, compared to comparison cohort, those with pneumoconiosis had multivariate HR (95% CI) for AF of 1.21 (1.08‐1.35) (Table 2).

**Table 2:** Incidence and hazard ratio of atrial fibrillation between patients with and without pneumoconiosis

	Pneumoconiosis							
	Yes			No				
Outcome	Event	PY	Rate^a^	Event	PY	Rate^a^	Crude HR^b^ (95% CI)	Adjusted HR^c^ (95% CI)
All	804	99 127	81.1	699	106 986	65.3	1.25 (1.13, 1.39)***	1.30 (1.17, 1.44)***
Gender								
Female	81	13 188	61.4	78	13 429	58.1	1.06 (0.78, 1.45)	1.10 (0.80, 1.50)
Male	723	85 939	84.1	621	93 556	66.4	1.28 (1.15, 1.43)***	1.33 (1.19, 1.48)***
Age, year								
≤64	147	34 753	42.3	103	36 953	27.9	1.54 (1.19, 1.97)***	1.58 (1.23, 2.03)***
65‐74	463	52 733	87.8	405	56 383	71.8	1.24 (1.08, 1.41)**	1.26 (1.10, 1.44)***
≥75	194	11 642	166.7	191	13 649	139.9	1.21 (0.99, 1.48)	1.19 (0.97, 1.45)
Comorbidity^d^								
No	182	23 017	79.1	119	24 683	48.2	1.64 (1.30, 2.06)***	1.70 (1.35, 2.14)***
Yes	622	76 110	81.7	580	82 302	70.5	1.17 (1.05, 1.32)**	1.21 (1.08, 1.35)**

Abbreviation: PY, person years.

a Incidence rate per 10 000 person‐years.

b Relative hazard ratio.

c Adjusted for age, sex, and all comorbidities listed in Table 1.

d Patients with any one of the following comorbidities were classified as the comorbidity group: hypertension, diabetes mellitus, hyperlipidemia, coronary artery disease (CAD), peripheral arterial occlusive disease (PAOD), chronic obstructive pulmonary disease (COPD), heart failure, asthma, interstitial lung disease, bronchiectasis, tuberculosis, liver cirrhosis, cancer, chronic kidney disease, hyperthyroidism, gout, sleep disorders, and stroke.

**P < .01; ***P < .001.

## 4 DISCUSSION

This retrospective study tried to investigate whether pneumoconiosis and AF are associated, and turned up with a positive answer. Patients with pneumoconiosis had a 30% higher risk of incident AF at a mean follow‐up of 8 years; this remained significant after adjustment for potential confounders by multivariable cox proportional hazard model analysis. The greatest strength of this study is its sample size. Moreover, the study methodology is reasonable, the follow‐up data was relatively robust, making this finding reliable. The results may provide an original contribution on the risk factors for AF development.

Based upon our results, this novel finding serves to generate an interesting hypothesis for further investigation. The most important question that this study raises is the relevance of this finding to the mechanism of AF. Some might argue that perhaps pneumoconiosis results in a greater vascular insult that may potentiate AF,^9^, ^10^, ^11^ or this group identifies a sicker cohort of patients that are more predisposed to development of AF. However, based upon our findings the association between pneumoconiosis and AF is even stronger in the subgroup of no comorbidity after adjustment for the covariates. Furthermore, both cohorts were relatively similar in terms of the prevalence of underlying medical comorbidities. In teasing out these distinctions, it might also reflect on the potential role that pneumoconiosis may play in atrial remodeling. Lung diseases commonly coexist, mostly due to shared common risk factors.^15^, ^16^, ^17^, ^18^, ^19^, ^20^, ^21^ In clinical practice, however, we may overlook coexistence of lung diseases and the risk of incident AF.^15^, ^16^, ^17^, ^18^, ^19^, ^20^, ^21^, ^22^, ^23^, ^24^, ^25^ There is great evidence in place to link bronchiectasis, COPD, asthma, interstitial lung disease, and AF and the risk of events.^22^, ^23^, ^24^, ^25^ In this study, the risk ratio is more profound in the subgroup of male gender and younger age after adjusting for an exhaustive list of covariates; implying that these population deserves more attention and future studies are encouraged to explore the fundamental mechanistic hypothesis in light of the link between pneumoconiosis and AF.

## 5 LIMITATIONS

First, the apparent relationship could be related to presence of several unmeasured confounders or of ascertainment bias. Second, lack of information on body mass index, physical activity, and alcohol use, which are major risk factors for AF and could have played a role as well. Finally, the diagnosis of AF was not made by ECG or Holter. There is also no information on types of AF (paroxysmal, persistent, or permanent). Neither was the data on treatment performed (medication, pulmonary vein ablation, AV node ablation + pacemaker, flutter ablation) provided. Despite these study limitations which can probably attenuate generalization of the results presented here; our study, a novel and interesting real‐world observation, reports on an increased AF rate in patients with pneumoconiosis.

## 6 CONCLUSION

Based on our results, pneumoconiosis and incident AF are associated.

## CONFLICT OF INTEREST

The authors declare no potential conflict of interests.
